# B-cell acute lymphoblastic leukemia promotes an immune suppressive microenvironment that can be overcome by IL-12

**DOI:** 10.1038/s41598-022-16152-z

**Published:** 2022-07-13

**Authors:** Rae Hunter, Kathleen J. Imbach, Chengjing Zhou, Jodi Dougan, Jamie A. G. Hamilton, Kevin Z. Chen, Priscilla Do, Ashley Townsel, Greg Gibson, Erik C. Dreaden, Edmund K. Waller, Karmella A. Haynes, Curtis J. Henry, Christopher C. Porter

**Affiliations:** 1 Cancer Biology Program, Laney Graduate School, Emory University, Atlanta, GA USA; 2 School of Biological Sciences, Georgia Institute of Technology, Atlanta, GA USA; 3 Department of Pediatrics, Emory University School of Medicine, Emory University, 2015 Uppergate Dr. NE, Rm. 433A, Atlanta, GA 30322 USA; 4 Aflac Cancer and Blood Disorders Center, Children’s Healthcare of Atlanta, 1760 Haygood Drive NE, Atlanta, GA 30322 USA; 5 Walter H. Coulter Department of Biomedical Engineering, Emory University and Georgia Institute of Technology, Atlanta, GA USA; 6 Winship Cancer Institute, Emory University, Atlanta, GA USA

**Keywords:** Acute lymphocytic leukaemia, Immune evasion, Cancer microenvironment

## Abstract

Immunotherapies have revolutionized the treatment of B-cell acute lymphoblastic leukemia (B-ALL), but the duration of responses is still sub-optimal. We sought to identify mechanisms of immune suppression in B-ALL and strategies to overcome them. Plasma collected from children with B-ALL with measurable residual disease after induction chemotherapy showed differential cytokine expression, particularly IL-7, while single-cell RNA-sequencing revealed the expression of genes associated with immune exhaustion in immune cell subsets. We also found that the supernatant of leukemia cells suppressed T-cell function ex vivo. Modeling B-ALL in mice, we observed an altered tumor immune microenvironment, including compromised activation of T-cells and dendritic cells (DC). However, recombinant IL-12 (rIL-12) treatment of mice with B-ALL restored the levels of several pro-inflammatory cytokines and chemokines in the bone marrow and increased the number of splenic and bone marrow resident T-cells and DCs. RNA-sequencing of T-cells isolated from vehicle and rIL-12 treated mice with B-ALL revealed that the leukemia-induced increase in genes associated with exhaustion, including *Lag3*, *Tigit,* and *Il10,* was abrogated with rIL-12 treatment. In addition, the cytolytic capacity of T-cells co-cultured with B-ALL cells was enhanced when IL-12 and blinatumomab treatments were combined. Overall, these results demonstrate that the leukemia immune suppressive microenvironment can be restored with rIL-12 treatment which has direct therapeutic implications.

## Introduction

Acute lymphoblastic leukemia (ALL) is the most common cancer in children and remains a leading cause of illness related death^1^, primarily related to relapsed disease. Current treatment strategies include highly toxic chemotherapy delivered at the cusp of tolerability^2^. Engaging the immune system using chimeric antigen receptor (CAR) expressing T-cells or the bispecific antibody blinatumomab, is highly effective at inducing responses and has revolutionized treatment of relapsed disease. But both strategies have considerable limitations, most importantly sub-optimal duration of response^3,4^, highlighting the limitations of current strategies.

The development and progression of leukemia is, in part, due to the ability of the leukemia cells to evade immune elimination. Although some mechanisms of immune evasion are similar in both hematological and solid cancers, leukemia and lymphoma have unique methods of evading the immune system^5,6^. Leukemia is known to alter the cellular and soluble composition of the BM^7–11^. Lack of antigen presentation and processing have been shown to contribute to the regulation of immune-cell tolerance in hematological malignancies^5,6^. Further, overexpression of PD-L1 co-inhibitory ligand on leukemia cells and changes in both immunostimulatory and immune suppressive cytokines have been shown to inhibit cytotoxic T-cells that are significant for clearance of leukemia^12–14^. Nonetheless, how leukemia cells evade the immune system remains incompletely understood.

Thus, we sought to better understand the molecular and cellular mechanisms of leukemia cell-mediated immune evasion. We first defined the tumor immune microenvironment in children with B-ALL by measuring circulating cytokines at the time of diagnosis, as well as single-cell RNA sequencing of non-leukemia cells. We observed differential cytokine expression in those with and without measurable residual disease (MRD) after induction chemotherapy and a gene expression profile consistent with immune exhaustion in those with MRD. Modelling T-cell function ex vivo, we observed a reduction in T-cell activation markers, CD44 and CD107a, on effector T-cells cultured in supernatant from human and murine B-ALL cell lines. We found further evidence of leukemia-induced immune suppression in a murine model of B-ALL that closely resembles human disease. Treating leukemia-bearing mice with rIL-12 restored T-cell numbers in the BM, consistent with our previous findings^15^. Furthermore, the number and activation state of dendritic cells (DCs) was also increased with rIL-12 treatment. In addition, rIL-12 treatment also altered levels of immunostimulatory cytokines and chemokines in the bone marrow. With targeted RNA-sequencing, we found upregulation of immune exhaustion genes in T-cells, including *Lag3*, *Tigit*, and *Il10,* in mice with leukemia compared to those without. Lastly, to determine the clinical implications of our findings, we determined if rIL-12 treatment impacted the efficacy of the immunotherapy, blinatumomab. In these studies, we found that B-ALL conditioned media attenuated the cytolytic capacity of T-cells when co-cultured with leukemia cells in the presence of blinatumomab, whereas treatment with rIL12 augmented the efficacy of blinatumomab in this condition. Together, these data provide mechanistic insight into B-ALL induced immunosuppression and highlight the therapeutic potential of IL-12 as a novel treatment for this disease.

## Materials and methods

### Single Cell RNA-sequencing

Peripheral blood or bone marrow from de-identified children with B-ALL was collected at the time of diagnosis after informed consent for biobanking. All experiments using human samples were approved by the Emory University Institutional Review Board (IRB# 00,034,535 and 00,089,506), in accordance with relevant guidelines and regulations. All samples were collected after informed consent of the subject or their parent/legal guardian. Frozen, Ficoll-separated samples from 4 children with MRD and 3 without MRD were available for analysis. Samples were sorted by flow cytometry for CD19^+^CD10^+^ lymphoblasts or CD45^+^CD19^neg^CD10^neg^ non-leukemia cells. The transcript data was processed using the Seurat package in R® statistical software. Quality control was implemented on the samples to retain cells only with transcripts for more than 200 unique genes and less than 30% mitochondrial contribution. All samples, both leukemic and nonleukemic, were then combined into one data object to ensure that flow cytometry had effectively separated malignant cells from peripheral immune cells^16,17^. The combined data object was log-normalized and scaled using the most highly variable genes in the dataset. Principal component analysis (PCA) was performed, and Shared Nearest Neighbor (SNN) analysis was implemented in Seurat’s FindNeighbors function, followed by a Uniform Manifold Approximation and Projection (UMAP) dimensional reduction technique, both using a dimensions parameter of 1:35. Finally, a UMAP plot was constructed, and clusters were visually separated. The groups of putative non-leukemia cells that mapped with the leukemia clusters were relabeled to reflect their true malignant identities. The few leukemia cells that mapped to the immune cell regions were omitted from downstream analyses. Once the cells were labeled to reflect their true malignancy states, analyses of non-leukemia and leukemia cell populations were performed separately. A total of 18,974 single cells were retained for further analysis: 5,906 nonleukemic immune cells and 13,068 leukemic cells (10X Genomics). Two methods were used to assign each cell a score based on their expression of exhaustion-specific marker genes. The first was calculated by totaling the normalized expression values for each of the exhaustion genes for each cell. The second resulted from the summed raw number of exhaustion gene transcripts per cell, normalized by the total transcripts per cell. In each case, the distribution of scores across all cells was assessed, and cells having upper-outlier score values in the two distributions were noted and labeled as highly exhausted cells. Genes included in the exhaustion score were *PDCD1, CTLA4, HAVCR2, TIGIT, TOX, LAG3, NFATC1, NFATC2, NR4A1, TOX2, TCF7, CD244, CD160,* and *ICOS*^18^.

### Mice

C57BL/6, and Rag1^-/-^ (B6.129S7-Rag1tm1Mom/J) mice were housed in microisolators in standard conditions at Emory University (Atlanta, GA). All experimental protocols and methods reported here have been carried out in accordance with rules on animal welfare and regulations under the ethical approval by Emory University Institutional Animal Care and Use Committee (IACUC). Animal studies were designed, executed and reported consistent with ARRIVE guidelines.

### Leukemia model

The luciferase-expressing, BCR-ABL1 Arf^-/-^ B-ALL cell line was originally provided by Dr. Richard Williams (St. Jude Children's Research Hospital, Memphis, TN)^19,20^. Cells were transduced with MSCV-iresGFP and sorted for GFP expression for some experiments. For in vivo experiments, a total of 2 × 10^5^ cells were transferred via tail vein injection into unirradiated, 6- to 8-week-old, female, wild-type (WT) C57BL/6 mice, with 3 mice per group (mice were randomly allocated to groups of mice without leukemia, vehicle treated mice with leukemia and rIL12 treated mice with leukemia). After intraperitoneal injection of luciferin and anesthesia with inhaled isoflurane, leukemia burden was measured by the In Vivo Imaging System (IVIS; Perkin Elmer). Mice were removed from the study and euthanized (per IACUC approved procedures) when ill-appearing or the luciferase signal exceeded 10^8^ photons/second, unless otherwise specified. Recombinant murine IL12-p70 was purchased from PeproTech and administered, unblinded, by intraperitoneal injection (1 μg/dose) daily for 5 days, beginning 3 days after leukemia transfer.

### Ex vivo leukemia cell culture

REH, RCH-ACV1 and NALM6 cell lines were acquired from ATCC. Leukemia cell lines were cultured in RPMI medium + 10% FBS + 1% penicillin/streptomycin + 0.1% 2-ME in a 37 °C incubator. Cells were plated at 0.5–2 × 10^5^ cells/ml and split every 48–72 h. For B-ALL supernatant collection, leukemia cells were plated at 5 × 10^5^ cells/ml and cultured for 24 h before collection of supernatant. Normal B-cell supernatant was collected after CD19^+^ cells were negatively selected from PBMCs via magnetic separation (Miltenyi Biotech, CD19 Microbeads, Cat#130–050-301) and incubated for 24 h with 5 ug/mL IgM (Abcam).

### T-cell activation and cytotoxicity assay

Human PBMCs were isolated from de-identified, normal donor buffy coats purchased from ZenBio (Durham, NC) or the Clinical and Translational Discovery Core (CTDC) at Children’s Healthcare of Atlanta using a Ficoll-density gradient. CD3^+^ T-cells were positively selected from PBMCs via magnetic separation (Miltenyi Biotech, CD3 Microbeads, Cat#130–050-101). T-cell purity was assessed at > 90% CD3^+^ of live cells, and the cells were cryopreserved. Cells were cultured in RPMI (10% FBS, 100 U/mL penicillin, 100 μg/mL streptomycin) at 37 °C, 5% CO2 unless otherwise specified. For T-cell activation experiments, CD3^+^ T-cells were stained with tracking dye CellTrace Yellow (ThermoFisher) prior to culture. After rest, T- cells were then plated in a 96-well u-bottom plate at 3.5 × 10^5^ cells/well. Plated cells were then stimulated with either stimulation cocktail (eBioscience) or Dynabeads™ Human T-Activator CD3/CD28 (ThermoFisher). On day 2 of culture, cells were harvested, and magnetic beads removed. Cells were then stained with CD44 APC (IM7, eBioscience), CD107a Pe-Cy5 (H4A3, BioLegend), and Live Dead Violet (Invitrogen) for 15 min in 4° in the dark. Cells were then washed twice with FACS buffer. For cytotoxicity experiments, Nalm-6 and CD3^+^ T-cells were stained with tracking dyes CellTrace Violet (Thermo) and CellTrace Yellow (Thermo), respectively, prior to culture. Nalm6 cells were then plated in a 96-well u-bottom plate at a 1:1 E:T ratio in RPMI-1640 (10% FBS) or Nalm6 supernatant and treated with vehicle (PBS) or 0.5 ng/mL-1 ng/mL blinatumomab (Invivogen, bimab-hcd19cd3) and/or 5–20 ng/mL rIL-12p70 (PeproTech) for 72 h. The cells were then prepared for flow cytometry. Live/Dead APC dye (Invitrogen) used to identify dead Nalm-6 cells.

Murine T-cells were obtained from splenocytes of C57BL/6 mice using EasySep™ Mouse T-cell Isolation kit (STEMCELL Technologies) or CD4/CD8 Microbeads (Miltenyi Biotech). CD3^+^ T-cells were stained with tracking dye CellTrace Yellow (ThermoFisher) prior to culture. After rest, T-cells were then plated in a 96-well u-bottom plate at 1 × 10^6^ cells/well. Plated cells were then stimulated with either stimulation cocktail (eBioscience) or murine Dynabeads™ Mouse T-Activator CD3/CD28 (ThermoFisher). On day 2 of culture, cells were harvested, the magnetic beads were removed, and cells were stained for flow cytometry.

### Flow cytometry

Samples were collected on the Cytek Aurora or Cytoflex flow cytometer (Beckman Coulter) and analyzed using FlowJo software (Tree Star). Gating strategies are shown in Supplemental Fig. 1A, B. Murine bone marrow samples from in vivo and in vitro experiments were acquired and stained using the following antibodies: anti–mouse CD16/32 (2.4G2; Fc block) from BD Biosciences; anti-mouse CD107b Alexa Fluor^®^ 647 (M3/84), CD8a BV510 (SK1), CD3ε Alexa Fluor^®^ 700 (500A2), CD11c APC (N418), CD4 APC/Fire™ 810 (GK1.5), CD127 Brilliant Violet 421™ (A7R34), CD80 Brilliant Violet 510™ (16-10A1), Ly-6C Brilliant Violet 570™ (HK1.4), CD86 Brilliant Violet 605™ (GL-1), Brilliant CD107a Violet 711™ (1D4B), CD11b Brilliant Violet 750™(M1/70), CD62L Brilliant Violet 785™ MEL-12), CD45R/B220 Pacific Blue™ (RA3-6B2), CD279 PE anti-mouse (29F.1A12), CD274 PE/Cyanine7(10F.9G2), Ly-6G PE/Dazzle™ 594 (1A8), KLRG1 PE/Dazzle™ 594 (2F1/KLRG1), CD44 PerCP/Cyanine5.5 (IM7), CD107b BV650 (CD8a Spark Blue™ 550 (53–6.7), Zombie NIR™ Fixable Viability Kit, all from BioLegend; anti–mouse MHC Class II (I-A/I-E) PerCP-eFluor 710 (M5/114.15.2), TIGIT PE/Dazzle™ 594 (1G9), LAG3 Brilliant Violet 650™(11C3C65), CD44 APC (IM7), all from eBioscience; CD107b APC-Vio770 (M3/84) from Miltenyi Biotec. Primary human T-cells were stained for purity post-isolation using anti-CD3 (OKT3, BioLegend), and Near IR Live/dead stain (Invitrogen).

**Figure 1:**
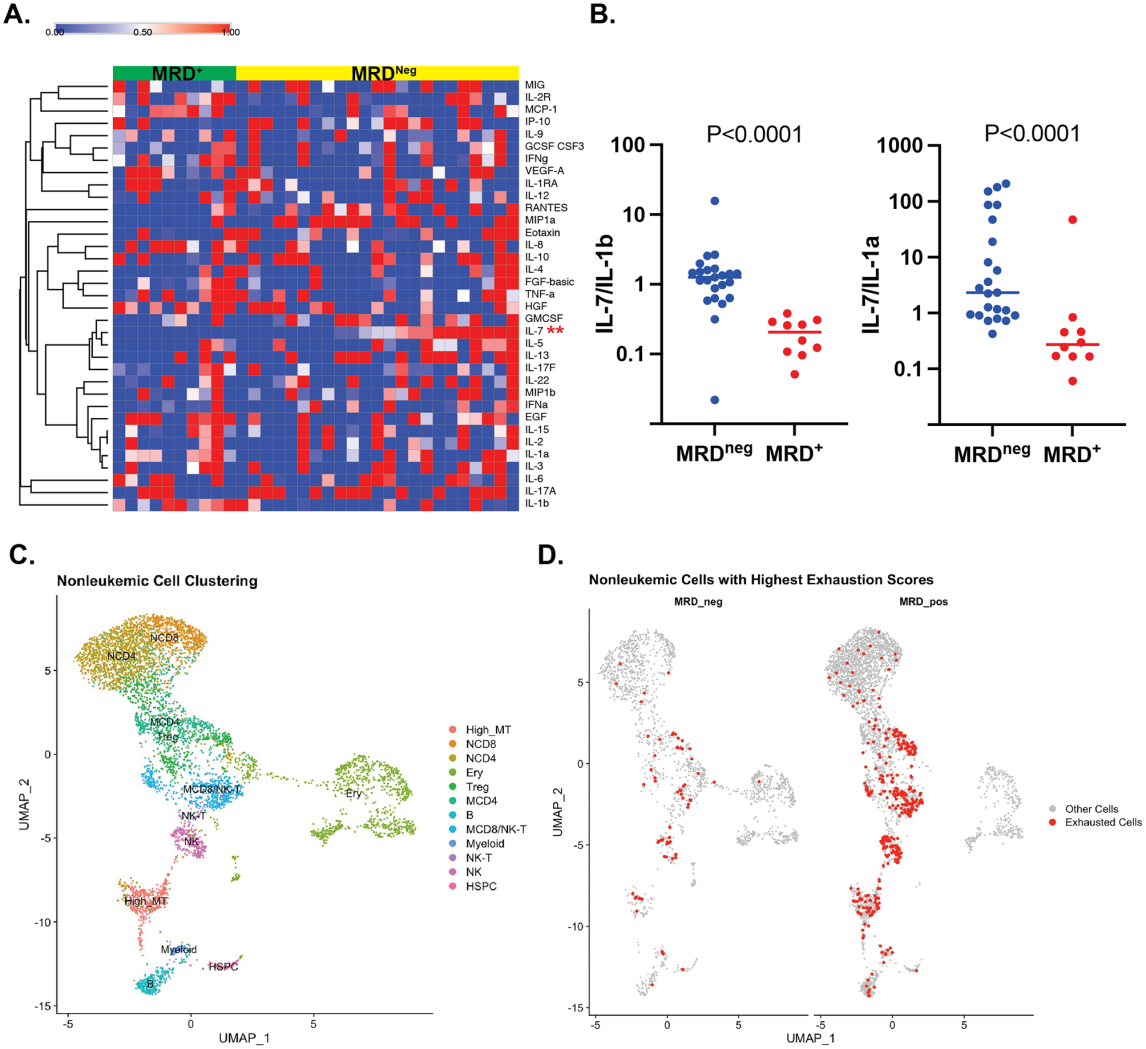
B-cell ALL alters the immune microenvironment. (**A**) Cytokines were measured via Luminex from the plasma of peripheral blood of children with B-ALL at the time of diagnosis. Cytokines are depicted in the heatmap relative to the median with supervised hierarchical clustering by residual disease at the end of induction chemotherapy (**P < 0.001 t test; FDR q value = 0.002; two-stage step up (Benjamini, Krieger & Yekutieli)). (**B**) The ratio of IL-7 to IL-1a and IL-1b is depicted (Mann Whitney test). (**C**) Dimensional reduction of non-leukemia cell populations using UMAP with cells colored and labeled by immune cell assignment. (**D**) Non-leukemia cells UMAP plot, split by disease outcome, with high exhaustion-scoring cells highlighted with high exhaustion scores in several cell populations including mature CD8 (MCD8) T-cells and natural killer T-cells (NK-T). Pearson's Chi-squared test with Yates' continuity correction used for statistical analysis demonstrates association of cells with high exhaustion score and MRD at end of induction chemotherapy (*p*-value < 5.2e-13).

### Cytokine ELISAs and luminex assays

Plasma from children with B-ALL was analyzed for cytokine and chemokine concentrations using the Cytokine 35-Plex Human Panel (ThermoFisher) for the Luminex platform. Supernatant acquired from T-cells cultured in vitro at a cell density of 1 × 10^6^ cells/well (murine) or 3.5 × 10^5^ cells/well (human) were collected after 48 h. The concentration of interferon-gamma (IFN- γ) in the supernatants was determined using either the mouse or human IFN-γ ELISA kit (RayBiotech; cat no. ELH-IFNg-1) per the manufacturer’s instructions. Absorbance at 450 nm was recorded using the Synergy 2 multi-mode microplate reader (Biotek). Murine bone marrow supernatant was harvested on day 7 post-engraftment from WT mice treated with rIL-12p70 or untreated for 5 days. Bone marrow serum was acquired in 0.5% BSA in PBS at 500 µl was analyzed by Eve Technologies (Calgary, AB, Canada) utilizing mouse cytokine and chemokine array 44-plex at onefold dilution.

### Quantitative gene expression analysis

Mouse bone marrow and spleen was harvested seven days after transplantation of luciferase–expressing BCR-ABL1^+^ B-ALL cells transduced with GFP. Pan-T-cell kit (Miltenyi Biotec) was used to isolate murine T-cells from the bone marrow of mice. RNA isolation and sequencing was performed by the Integrated Genomics Core at Emory University using nCounter® Immune Exhaustion Panel (nanoString, Seattle, WA) that profiles 785 genes across 47 pathways. Data was normalized using Nsolver database and log10 fold change was assessed for heat map generation. Protein–protein interaction (Ppi) differences were imported from STRING (https://string-db.org/) into Cytoscape using the list of Uniprot IDs (crosschecked with STRING as needed).

### Statistical analysis

Most statistical analyses were performed using GraphPad Prism software. Statistical significance between two groups was determined by a Student’s t-test, while ANOVA with Tukey multiple comparison test was used to test significance between three or more groups. Error bars in figures represent the SD and may be obscured when narrow. Animal experiments included at least 3 mice/group, and data from all mice are included. To minimize animal use, in vivo experiments were repeated only once. A false discovery rate (FDR) with adjusted *p*-value of 0.05 was used to generate volcano plots. Gene ontology data was generated based on at least two-fold up- or down- regulation compared to the no leukemia control, and *p*-value < 0.05. Heatmaps and hierarchical clustering were generated using Morpheus (https://software.broadinstitute.org/morpheus). The datasets generated are available in the NCBI GEO data repository (GSE198519).

## Results

### Changes in the immune microenvironment of B-ALL at the time of diagnosis are associated with MRD

The immune microenvironment is both influenced by and influences the development of B-ALL^7–11^. As MRD at the end of induction chemotherapy is the strongest predictor of relapse in children with B-ALL^2^, we sought to compare the immune microenvironment at the time of diagnosis in those with and without MRD. We first measured a panel of cytokines and chemokines in the plasma in children with B-ALL. After correction for multiple comparisons, among the analytes measured and reliably detected, only IL-7 was detected at different levels, with higher levels in those without MRD (Fig. 1A). In addition, the ratio of IL-7 to both IL-1α and IL-1β was significantly higher in those without MRD (Fig. 1B).

To determine the impact of MRD on the cellular immune microenvironment, we focused analyses of single-cell RNA-sequencing (scRNA-seq) on non-malignant immune cells derived from pediatric patients with B-ALL. Cells were clustered based on the expression of genes associated with specific hematopoietic subsets (Fig. 1C). Among, the defined subsets, only the erythroid precursor population was significantly associated with outcome, with greater percentages of erythroid precursors in those without MRD (38.7% vs. 7.3%, *P* < 0.0001, 2-way ANOVA with Sidak’s multiple comparison test). We next focused on genes involved in immune exhaustion and identified more cells with high immune exhaustion scores in those with MRD at the end of induction therapy, including T-cells and natural killer (NK) cells, which are important for eradicating leukemia cells (Fig. 1D). These results demonstrate that the presence of residual disease coincides with immune dysfunction at the time of diagnosis, notably cytotoxic cells with exhausted gene expression signatures.

### The B-ALL secretome suppresses T-cell activation

**Figure 2:**
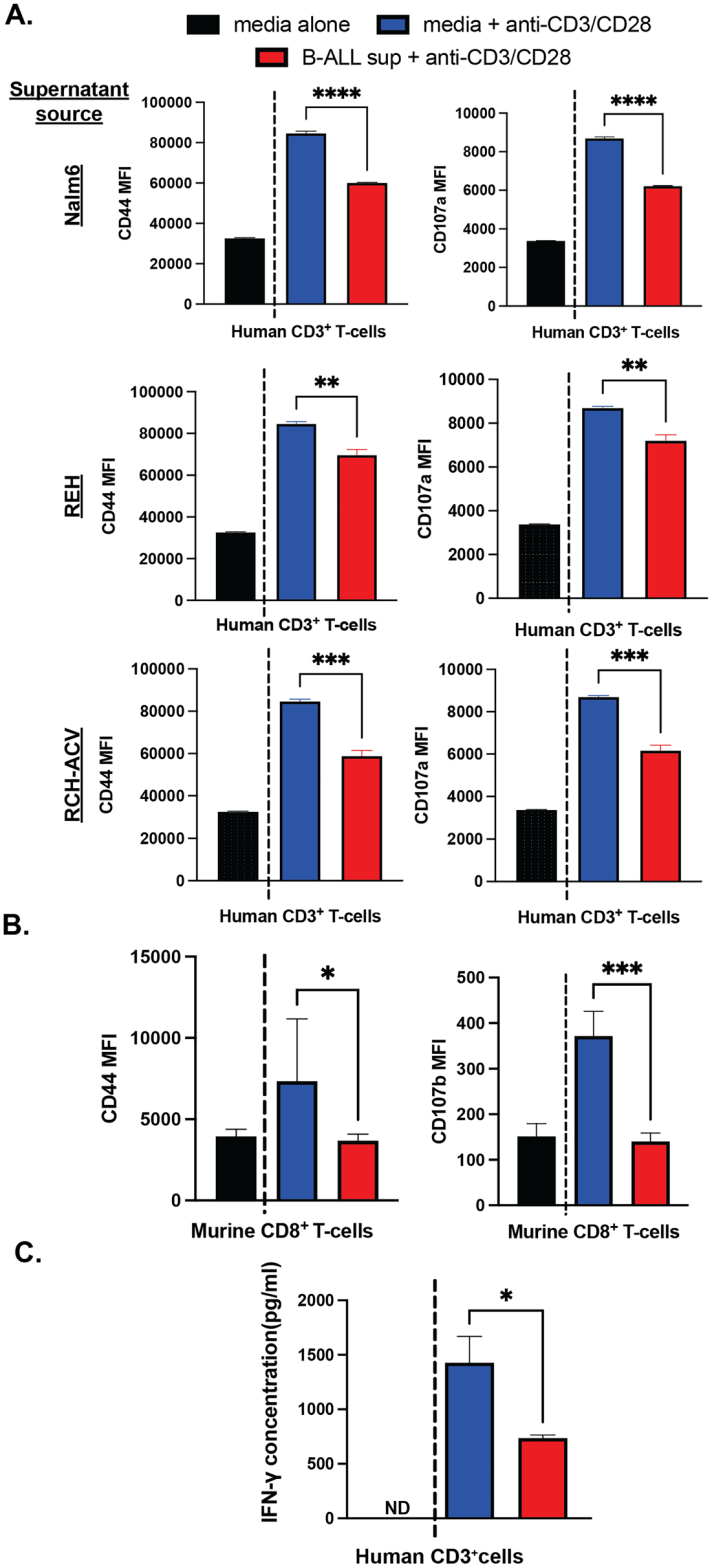
The B-ALL secretome suppresses T-cell activation*.* (**A**) CD3^+^ human T-cells stimulated ex vivo with CD3/CD28 antibodies were co-cultured in either Nalm6, REH or Rch-Acv supernatant for 48 h and analyzed via flow cytometry. Graphs depict mean fluorescence intensity (MFI) for CD44 (left) and CD107a (right) antibodies (*n* = 2 independent experiments; *P < 0.05; **P < 0.01; ****P < 0.0001, ANOVA with Tukey multiple comparisons test) (**B**) Murine splenocytes were stimulated ex vivo with CD3/CD28 antibodies and cultured with B-ALL supernatant (Nalm6) and analyzed by flow cytometry (*n* = 3 independent experiments; *P < 0.05; **P < 0.01; ****P < 0.0001, ANOVA with Tukey multiple comparisons test). (**C**) ELISA for IFN-γ from supernatant of human T-cells cultured in B-ALL conditioned media.

Based on these results, we next determined how B-ALL cells directly impact T-cell activation. Human CD3^+^ T-cells were stimulated in vitro with either anti-CD3/CD28 and cultured in unconditioned media or conditioned media derived from the supernatants of Nalm-6, REH, or RCH-AcV human B-ALL cell lines. Human T-cells stimulated in B-ALL supernatants expressed significantly lower levels of surface markers associated with T-cell activation including CD44 (which plays a role in T-cell adhesion^21^) and CD107a (a lysosomal protein transported to the T-cell surface during the degranulation of cytolytic content^22,23^; Fig. 2A). Similarly, the activation of murine T-cells was suppressed by the B-ALL secretome, resulting in lower surface expression of CD44 and CD107b expression (another surface marker of degranulation^24^; Fig. 2B). Notably, supernatant from stimulated normal B-cells did not suppress T-cell activation (Supplemental Fig. 2), indicating this characteristic is specific to transformed cells. In addition to the suppression of surface proteins associated with T-cell activation, the production of effector cytokines by human CD3^+^ T-cells, notably IFN-γ, was significantly inhibited by B-ALL secreted factors (Fig. 2C). These data supported our scRNA-seq results demonstrating that T-cell suppression occurs in the B-ALL microenvironment and provided direct evidence that the B-ALL secretome potently suppresses T-cell effector function.

### B-ALL induces cellular changes in the B-ALL microenvironment that can be normalized by IL-12

**Figure 3:**
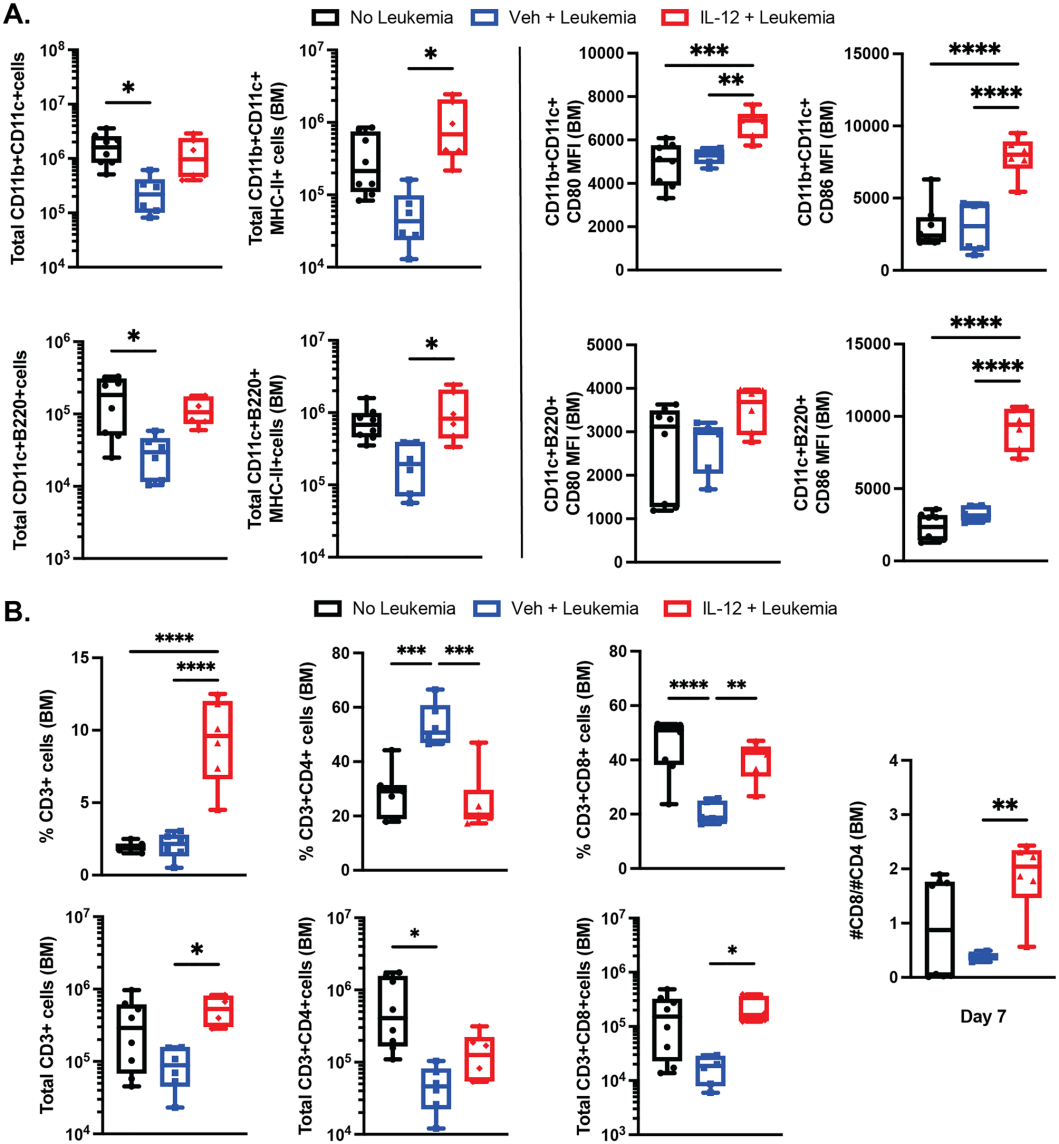
B-ALL induces cellular changes in the B-ALL microenvironment that can be normalized by IL-12. Parental BCR-ABL1^+^/Arf^−/−^ leukemia cells were injected into unirradiated WT recipients. On day 3, treatment with either rIL12 or BSA was begun (1 μg/dose, intraperitoneal, on days 3–7, 10–12); *n* = 6/group from two independent experiments). (**A**) Total CD11b^+^CD11c^+^ cells and CD11c^+^B220^+^ cells, as well as total MHCII^+^ cells and CD86 and CD80 mean fluorescence intensity (MFI) of each cell population, respectively. (**B**) The percentage (top) and total number (bottom) of specified T-cell populations in the bone marrow. The ratio of the number of CD8 to CD4 cells is also shown (right). (ANOVA with Tukey multiple comparison test).

To model immune microenvironment changes in vivo, we used a well-characterized mouse leukemia, driven by BCR-ABL1, that rapidly engrafts and progresses lethally in non-irradiated, immune competent recipients^19,20^. With this model, we previously demonstrated that IL-12 promotes T-cell dependent immune clearance of leukemia and prolongs survival. Furthermore, we demonstrated that protection correlated with changes in T-cell subset numbers in the bone marrow^15^. When BM cells were harvested from mice 7 days after engraftment of leukemia cells, we found alterations in the number and activation state of several immune cell populations. Similar to previous observations in humans^7^, the most consistent leukemia-induced changes in the immune microenvironment were in the myeloid compartment (Fig. 3A). Specifically, we observed significant reductions in the total number of conventional (CD11c^+^CD11b^+^) and plasmacytoid (CD11c^+^B220^+^) dendritic cells. Treatment of the mice with leukemia using recombinant IL-12p70 (rIL-12) restored the numbers of these cells and enhanced the activation state of both conventional and plasmacytoid DCs, which expressed higher surface levels of T-cell costimulatory molecules (CD80 and CD86) and MHC class II molecules which present antigens to CD4^+^ T-cells (Fig. 3A). In addition, mice with leukemia had changes in the T-cell compartment, with higher proportions of CD4^+^ cells and lower proportions of CD8^+^ cells (Fig. 3B). However, treatment with rIL-12 increased the percentage of T-cells in the bone marrow, such that the number of T-cells was restored, as was the ratio of CD8^+^/CD4^+^ cells (Fig. 3B). As before, rIL-12 treatment of B-ALL-bearing mice significantly reduced leukemia burden at day 10 post-transplantation of leukemia cells (Supplemental Fig. 3). Together, these data demonstrate extensive remodeling of the cellular tumor immune microenvironment in response to leukemia, which can be normalized to some extent by treatment with rIL-12, perhaps by heightened T-cell priming capacities by resident DC subsets.

### IL-12 treatment of B-ALL bearing mice creates an immunostimulatory soluble milieu in the bone marrow

**Figure 4:**
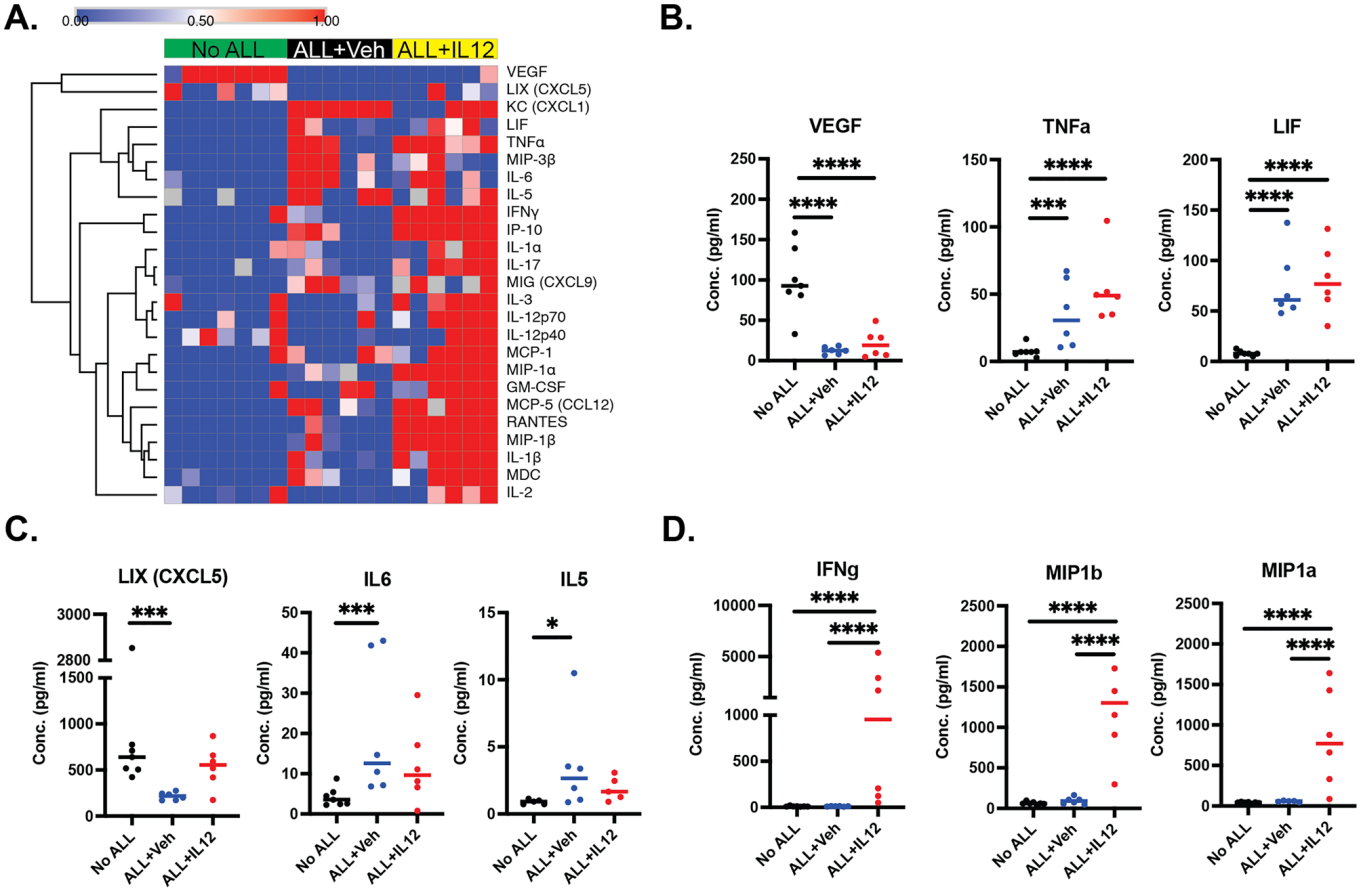
IL-12 treatment of B-ALL bearing mice creates an immunostimulatory soluble milieu in the bone marrow. (**A**) Luminex assay was used to measure cytokines and chemokines levels in the bone marrow of control and rIL-12 treated WT mice at day 7 (*n* = 6 mice/group). The heatmap shows concentrations relative to the median with supervised hierarchical clustering by treatment condition. (**B**) Cytokines with altered concentrations attributed to leukemia. (**C**) Cytokines/chemokines with altered concentrations due to leukemia and normalized with IL-12. (**D**) Cytokines/chemokines altered due to IL-12. (*P < 0.05; **P < 0.01; ****P < 0.0001, ANOVA with Tukey multiple comparisons test).

We next sought to determine the extent to which leukemia alters immune signaling systems in the bone marrow, as well as changes induced by rIL-12. To this end, we performed a multiplexed cytokine/chemokine assay from supernatant collected from the bone marrow 7 days after engraftment of leukemia. After excluding analytes that were not reliably detected, scaling for batch effects, and correcting for multiple comparisons, a few notable patterns of differential cytokine levels were of interest (Fig. 4A). First, was a set of cytokines/chemokines that was altered based on the presence of leukemia (Fig. 4B). Second was a set of analytes with differential levels due to leukemia but normalized by IL-12 (Fig. 4C). The most dramatic differences were seen in proinflammatory cytokines induced by rIL-12, including IFN-γ (Fig. 4D), consistent with a robust immune response to the leukemia cells. These results demonstrate that rIL-12 treatment of leukemia-bearing mice may augment anti-leukemia immunity by promoting an immunostimulatory cytokine/chemokine milieu.

### Genes associated with T-cell exhaustion are induced in B-ALL-bearing mice

**Figure 5:**
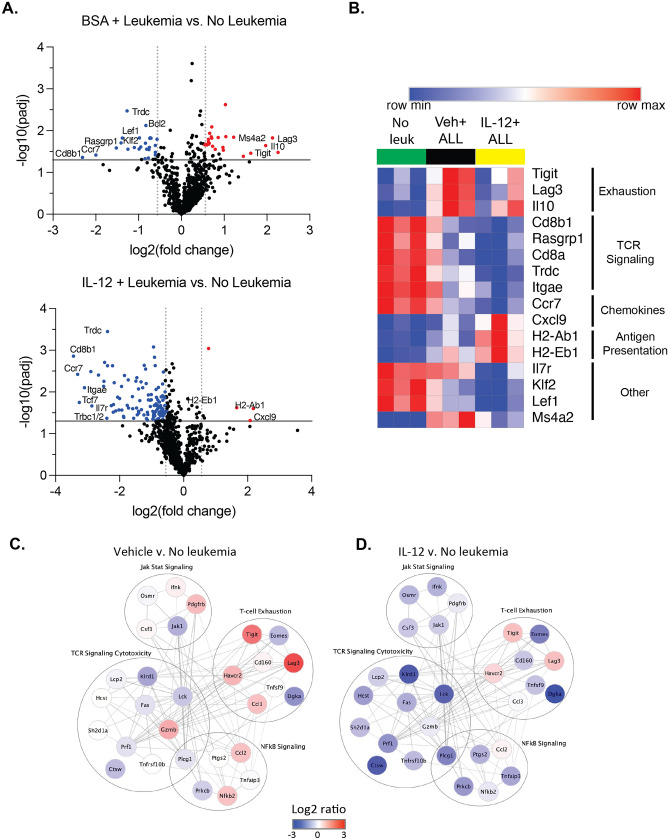
Genes associated with T-cell exhaustion are induced in B-ALL-bearing mice. Murine T-cells were isolated from the bone marrow of either untreated or rIL-12 treated WT mice at day 7 and subjected to targeted RNA sequencing using the nCounter Immune Exhaustion panel (Nanostring). (**A**) Volcano plots of gene expression changes in T-cells from vehicle treated (top) or IL-12 treated (bottom) mice compared to mice without leukemia. (**B**) Heat map demonstrating relative gene expression of indicated genes. (**C**,**D**) Protein–protein interaction networks with functional annotation from differentially expressed genes (FC >  = 1.5, *P* < 0.05).

As we previously demonstrated that T-cells are essential for effective immune clearance of leukemia cells^15^, we sought to determine the molecular mechanisms of T-cell failure and success by performing targeted gene expression profiles of T-cells from the leukemia immune microenvironment. T-cell populations from vehicle treated mice with leukemia exhibited significant upregulation of genes associated with immune exhaustion, such as *Lag3, Tigit* and *Il10*, as well as *Ms4a2*, an immune suppressive gene expressed by T-regulatory cells^25^. However, these genes were not as highly expressed in T-cells from rIL-12 treated mice with leukemia (Fig. 5A,B). In fact, the vast majority of differentially expressed genes in T-cells isolated from B-ALL bearing mice treated with rIL-12 were expressed at lower levels, as compared to T-cells from mice without leukemia, including some also downregulated in the vehicle treated mice with leukemia, suggesting leukemia-mediated suppression (Fig. 5A,B).

We then examined protein–protein interaction (Ppi) networks after grouping genes under functional annotations for our RNA-seq data, to identify how protein interactions in T-cells may be regulated under the conditions tested. This also demonstrated a trend in upregulation of genes associated with immune exhaustion in T-cells from leukemia-bearing relative to naïve mice (Fig. 5C), but to a lesser extent in those treated with IL-12 (Fig. 5D).

**Figure 6:**
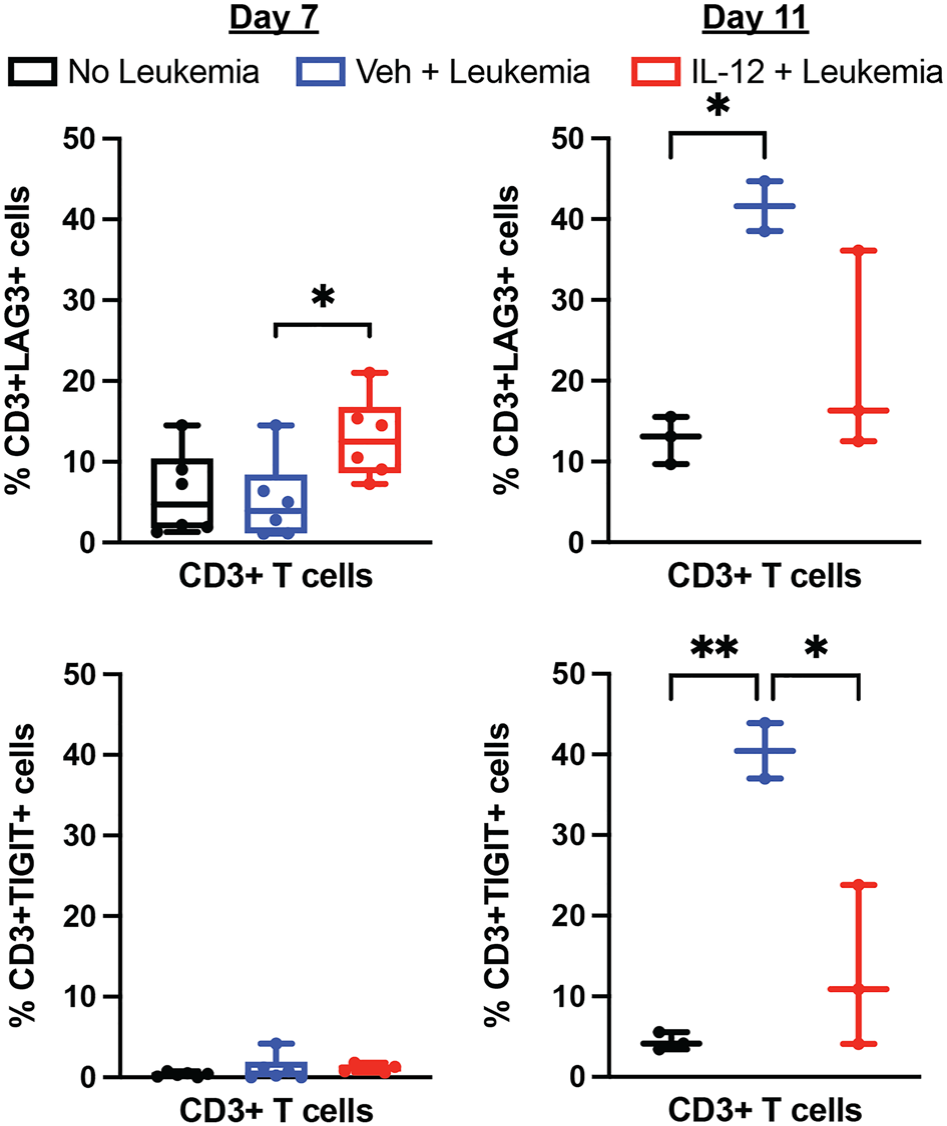
IL-12 abrogates leukemia-induced increases in cell surface expression of LAG3 and TIGIT on T-cells. Bone marrow cells were harvested from mice as in Fig. 3 on day 7 (left; *n* = 6/group) and day 11 (right; *n* = 3/group) for flow cytometry. The percentage of LAG3^+^ (top) and TIGIT^+^ (bottom) T-cells is demonstrated. (*P < 0.05, **P < 0.01; ANOVA with Tukey multiple comparisons test).

To determine if the gene expression changes of exhaustion markers were reflected in differential expression of protein at the cell surface, we performed additional flow cytometry of bone marrow from mice at 7 and 11 days after injection of leukemia cells. Staining for exhaustion markers LAG3 and TIGIT demonstrated an increase in expression on CD3^+^ T-cells in vehicle treated mice with leukemia between days 7 and 11, which is diminished by treatment with IL-12 (Fig. 6), generally consistent with the gene expression analyses. Together, these data demonstrate upregulation of several genes in T-cells, in response to leukemia, including those associated with immune exhaustion, which is abrogated by treatment with IL-12.

### Combining blinatumomab and IL-12 therapies overcomes B-ALL suppression of T-cells

**Figure 7:**
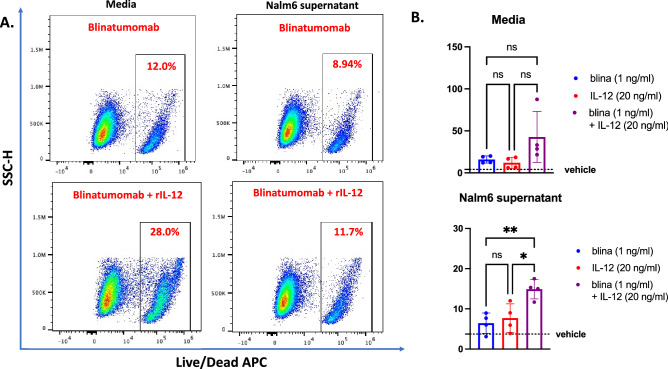
IL-12 overcomes reduced blinatumomab efficacy in the B-ALL secretome. (**A**) Representative flow cytometry plots of T-cell cytolytic activity showing leukemia cell death in different experimental conditions. (**B**) Leukemia cell death from T-cell cytolytic killing cultured in either control media (top panel) or Nalm6 supernatant (bottom panel) (*n* = 4 donors; *P < 0.05; **P < 0.01; ****P < 0.0001, ANOVA with Tukey multiple comparisons test).

Based on these observations, we determined if combining blinatumomab and rIL-12 treatments overcome B-ALL-mediated suppression of T-cells. Notably, we observed that B-ALL secreted factors compromised blinatumomab-mediated killing of B-ALL cells when co-cultured with CD3^+^ human T-cells (Fig. 7A, Supplemental Fig. 4). Interestingly, rIL-12 treatment overcame this suppression and resulted in a two-fold increase in the cytotoxicity of B-ALL cells when co-cultured with human T-cells and blinatumomab in leukemia cell supernatant (Fig. 7B).

In all, these data suggest that B-ALL mediated immunosuppression can be overcome with rIL-12 treatment, and this strategy may represent a novel approach to augment the efficacy blinatumomab in MRD-positive patients with B-ALL.

### Discussion

Leukemia is a leading cause of disease-related deaths in children, and immunotherapies have emerged as revolutionary treatments for patients with refractory or relapse disease. Despite best clinical efforts, immunotherapies targeting CD19-expressing malignant B-cells, notably blinatumomab and chimeric antigen receptor (CAR) T-cells, have failed to elicit long-term protection in many pediatric patients with relapsed disease^3, 4^ which highlights the need for novel strategies to optimize the efficacy of these groundbreaking treatments.

In these studies, we present data demonstrating that the presence of MRD in patients diagnosed with B-ALL is associated with an altered immune microenvironment. While a small study, to our knowledge, this is the first demonstration of altered immunity associated with MRD, the strongest predictor of relapse, which could be determined at the time of diagnosis and be used as earlier biomarkers of high-risk disease, if validated prospectively. IL-7 signaling promotes T-cell expansion and plays a key role in the development of memory T-cells^26^. In addition, IL-1β paradoxically enhances tumor progression by promoting an immune suppressive microenvironment with fewer IL-12 secreting dendritic cells^27^. While our models of B-ALL immune suppression were not designed to explore the role of individual cytokines, our in vitro and murine models of B-ALL demonstrated T-cell suppression and exhaustion directly induced by B-ALL-secreted factors and the leukemic microenvironment, respectively. Human and murine T-cells in both contexts exhibited attenuated T-cell effector responses (significantly lower CD44 surface levels and reduced IFN-γ production) and gene expression profiles and cell surface markers indicative of T-cell exhaustion (*Il10, Lag3, Tigit*)*.* T-cell exhaustion has been observed in murine models of ALL, in which B-ALL induces the expression of PD1, TIM3 and LAG3^28^, and pediatric cases of B-cell precursor acute lymphoblastic leukemia (BCP-ALL) where high numbers of TIM-3^+^ CD4^+^ T-cells is correlated with a higher risk of relapse^29^. Notably, we demonstrated that treatment with rIL-12 overcomes B-ALL-induced immunosuppression, highlighted by the establishment of an immunostimulatory leukemia microenvironment in the bone marrow, characterized by high levels of IFN-γ, IL-2, and various chemokines, higher numbers activated DC subsets in the bone marrow, and elevated numbers of highly functional CD8^+^ T-cells. Importantly, we provide data supporting the adjuvant potential of rIL-12 treatment as a novel approach to overcome B-ALL-mediated immunosuppression in the context of blinatumomab treatment, where we observed enhanced killing of malignant B-cells when co-cultured with human T-cells with the addition of rIL12. Taken together, these data demonstrate the pleiotropic immunostimulatory potential of rL-12 treatment as a mechanism to improve outcomes in settings of B-ALL.

IL-12 has shown remarkable anti-tumor efficacy in a wide range of malignancies in preclinical studies^30^. While most of these studies are in solid tumors^31–34^, we and others have demonstrated the potency of IL-12 in promoting immunologic elimination of leukemia cells, dependent upon both CD4^+^ and CD8^+^ T-cells, and to a lesser extent NK cells^15, 35^. To our knowledge, we are the first to report (1) T-cell suppression by B-ALL supernatant, and (2) the reversal of this suppression in bone marrow resident T-cells in leukemia bearing mice treated with rIL-12. Our findings support previous research on IL-12 as a co-stimulatory molecule that induces T_H_1 differentiation and increases activation and cytotoxicity of T-cells^36,37^. It also supports published animal model and clinical studies demonstrating IL-12-induced upregulation of pro-inflammatory mediators including IFN-γ from T-cells and the upregulation of MHC I and II surface expression on antigen presenting cells (APCs)^38–40^.

We also identified enhanced efficacy of blinatumomab with IL-12 treatment in T-cells exposed to B-ALL supernatant compared to blinatumomab treatment alone. Our results demonstrate the potential for IL-12 to be used as a therapeutic in B-ALL patients. However, despite demonstrating potential anti-tumor activity in preclinical studies, systemic administration of IL-12 was associated with limited benefit and severe adverse effects in clinical trials^41–43^. It should also be noted that our in vivo model has limitations, including the absence of immune suppression induced by chemotherapeutics, which may temper the effect of IL-12, as well as blinatumomab. Nonetheless, our results suggest that the targeted delivery of IL-12 and blinatumomab may be an effective strategy to overcome T-cell exhaustion associated with MRD (Fig. 1). In further support of this concept, we previously demonstrated that bi-specific T-cell engaging (αCD19:αCD3) nanoparticles formulated to also deliver IL-12 to the immune synapse (termed BiTEokines) provided superior killing of human B-ALL cells when co-cultured with human T-cells relative to single-agent treatment with blinatumomab or rIL-12^44^. In collaborative studies, we are currently in the process of testing the efficacy of BiTEokines in murine models of B-ALL.

In conclusion, our work demonstrates the potent immuno-rejuvenating potential of rIL-12 treatment in the context of B-ALL and demonstrates its potential as an adjuvant to improve the efficacy of blinatumomab treatment in patients with relapsed or refractory disease.

## Supplementary Information

Supplementary Information.
