# Supplementary material for: B-cell acute lymphoblastic leukemia promotes an immune suppressive microenvironment that can be overcome by IL-12
Source: Sci Rep. 2022 Jul 13;12:11870. doi: 10.1038/s41598-022-16152-z (PMC9279427; doi:10.1038/s41598-022-16152-z)

B-cell acute lymphoblastic leukemia promotes an immune suppressive microenvironment that can be overcome by IL-12

Rae Hunter, Kathleen Imbach, Chengjing Zhou, Jodi Dougan, Jamie A.G. Hamilton, Kevin Z. Chen, Priscilla Do, Ashley Townsel, Greg Gibson, Erik C. Dreaden, Edmund K. Waller, Karmella Haynes, Curtis J. Henry, Christopher C. Porter

Supplemental Figures

Supplemental Figure 1. A. Representative dot-plots of flow cytometry for $CD8^+$ and $CD4^+$ T-cells. **B.** Representative dot-plots of flow cytometry for $CD11b^+CD11c^+$ (conventional), $CD11c^+B220^+$ (plasmacytoid) dendritic cells (DC), MHC-II^+ cells in both DC populations, and activation markers, CD80 and CD86 expression in the BM (blue histogram – unstained control; red histogram – Ab stained).

A.

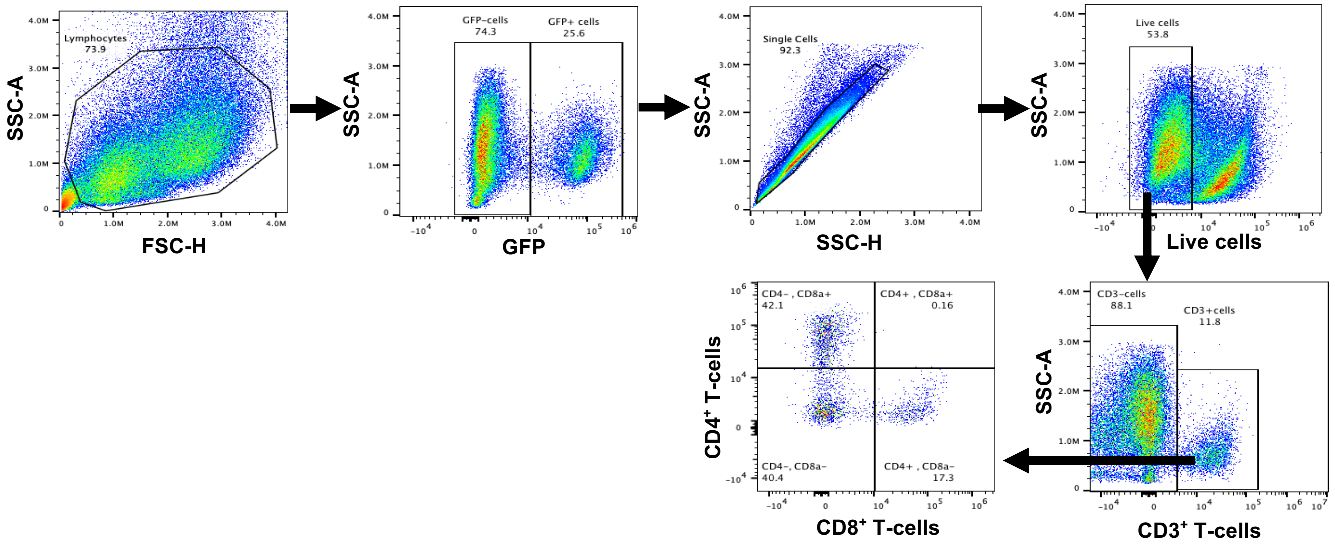

B.

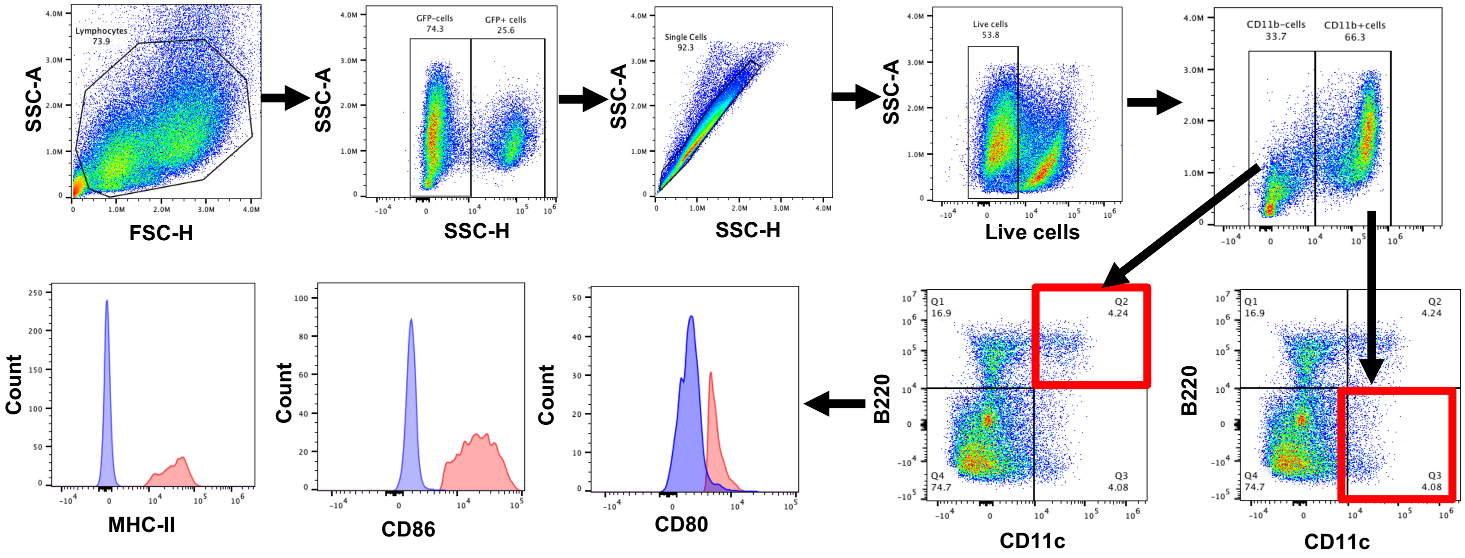

Supplemental Figure 2. Representative MFI quantification of CD44 and CD107a expression of three human donor T cells in either media or normal B-cell supernatant.

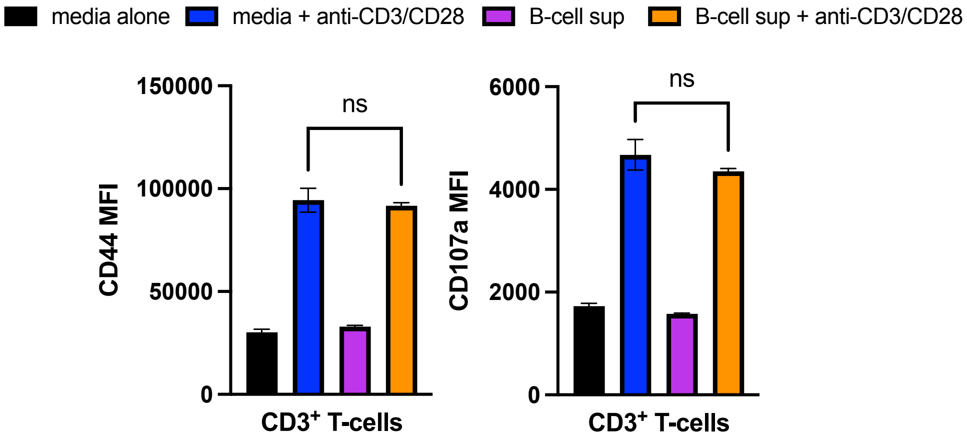

Supplemental Figure 3. Luciferase signal over time in WT recipients of B-ALL either untreated or treated with rIL-12 (n=6/group from 2 independent experiments).

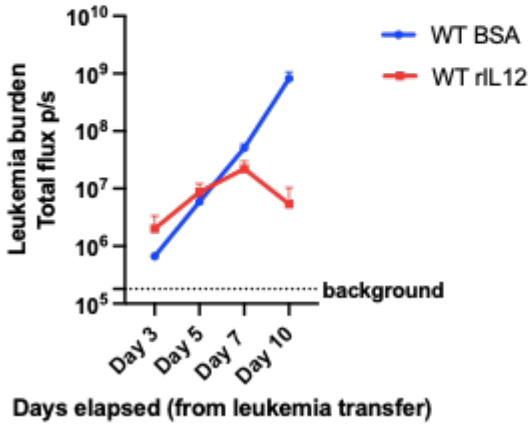

Supplemental Figure 4. Representative dot-plots of flow cytometry for leukemia cell lysis.

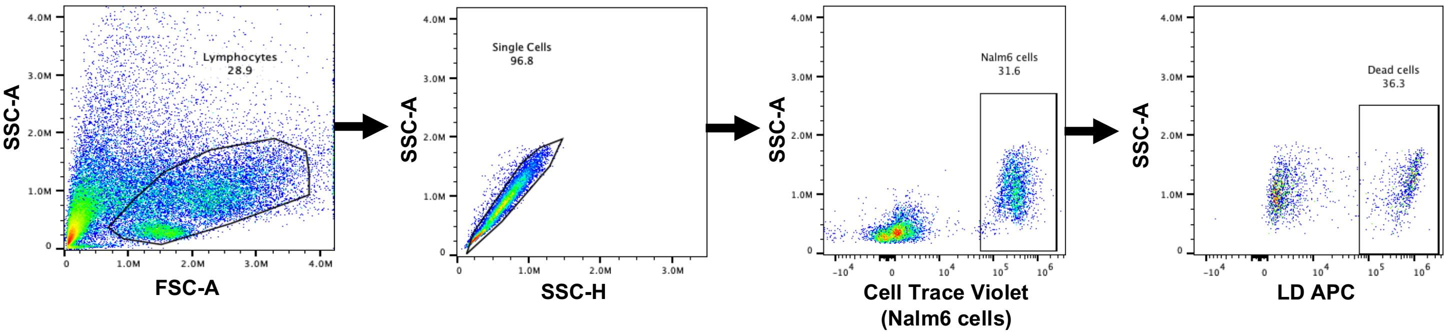

Supplement: Supplementary file 1 — Supplementary Information. [file 41598_2022_16152_MOESM1_ESM.pdf]
